# Rayleigh-wave dispersion reveals crust-mantle decoupling beneath eastern Tibet

**DOI:** 10.1038/srep16644

**Published:** 2015-11-09

**Authors:** Cédric P. Legendre, Frédéric Deschamps, Li Zhao, Qi-Fu Chen

**Affiliations:** 1 Institute of Earth Sciences, Academia Sinica, 128 Academia Road, Sec. 2, Nangang, Taipei 11529, Taiwan; 2 Key Laboratory of Earth and Planetary Physics, Institute of Geology and Geophysics, Chinese Academy of Sciences, Beijing 100029, China; 2 Present address: Department of Geosciences, National Taiwan University, No. 1, Sec 4, Roosevelt Rd., Taipei 10617, Taiwan

## Abstract

The Tibetan Plateau results from the collision of the Indian and Eurasian Plates during the Cenozoic, which produced at least 2,000 km of convergence. Its tectonics is dominated by an eastward extrusion of crustal material that has been explained by models implying either a mechanical decoupling between the crust and the lithosphere, or lithospheric deformation. Discriminating between these end-member models requires constraints on crustal and lithospheric mantle deformations. Distribution of seismic anisotropy may be inferred from the mapping of azimuthal anisotropy of surface waves. Here, we use data from the CNSN to map Rayleigh-wave azimuthal anisotropy in the crust and lithospheric mantle beneath eastern Tibet. Beneath Tibet, the anisotropic patterns at periods sampling the crust support an eastward flow up to 100°E in longitude, and a southward bend between 100°E and 104°E. At longer periods, sampling the lithospheric mantle, the anisotropic structures are consistent with the absolute plate motion. By contrast, in the Sino-Korean and Yangtze cratons, the direction of fast propagation remains unchanged throughout the period range sampling the crust and lithospheric mantle. These observations suggest that the crust and lithospheric mantle are mechanically decoupled beneath eastern Tibet, and coupled beneath the Sino-Korean and Yangtze cratons.

The convergence of Indian and Eurasian plates started ca. 50 Myr ago and resulted in the formation of the Tibetan Plateau. Since the beginning of this collision, the Tibetan crust has doubled its thickness^1^, and the Tibetan Plateau has been elevated by 4–5 km^2^,^3^. The main tectonic features of the Tibetan Plateau are summarized in Fig. 1. Within the plateau interior, the crust flows roughly eastward with speeds that increase toward the east, and then flows southward around the eastern end of the Himalaya^4^,^5^. Mechanisms invoked to explain this eastward extrusion imply major strike-slip faults^2^,^3^,^6^. Fission-track thermochronology^7^ and magnetotelluric imaging^8^ in this region further support the hypothesis that the eastward crustal flow plays a key role in the tectonics and deformation of the Tibetan Plateau. These deformation characteristics have been attributed to the ductile channel flow in a weak lower crust^9^,^10^,^11^. Crustal flow is not uniform beneath the southeastern Tibetan Plateau^12^, and it may be linked with mechanical conditions in the periphery. These observations are best explained assuming a mechanical decoupling between the crust and the lithospheric mantle. Despite the high elevation of this region, estimations of crustal thickening in the eastern margin of Tibet^4^,^5^,^9^,^13^ do not show strong evidence for significant shortening of the upper crust^14^. This observation was interpreted as the consequence of a ductile flow of the lower crust implying, again, a mechanical decoupling between the crust and lithospheric mantle^9^. Alternatively, it has been suggested that crustal deformation results from lithospheric flow as a whole, involving a mechanical coupling between the crust and the mantle. The mechanical coupling hypothesis is supported by comparisons between the observed surface deformation inferred from GPS and Quaternary fault slip rate data, and the mantle deformation inferred from several SKS shear-wave splitting datasets^15^,^16^, which suggest a coherence between crust and mantle deformation. Discriminating between the mechanical coupling or decoupling between crust and lithospheric mantle requires additional information that can constrain the deformation within the crust and lithospheric mantle.

Seismic data provides key information on the structure and deformation of the lithosphere. Seismic tomography allows imaging crustal fault systems and structures at depth, therefore bringing information on the regional dynamics. Regional traveltime tomography^17^,^18^ and surface-wave tomography^19^,^20^,^21^,^22^ displayed very low velocity anomalies in the crust and upper mantle in western Sichuan and in the Tengchong volcanic area, indicating a possible rotation of the crustal channel flow along the western edge of the rigid Sichuan Basin^9^,^10^,^11^. Because it can be related to rock deformation through lattice preferred orientation (LPO), seismic anisotropy is a key observation to understand the deformation of the Tibetan lithosphere. Shear-wave splitting studies^13^,^23^,^24^,^25^ have revealed a complex anisotropic pattern, with a dramatic change in the fast polarization direction across the Chuan-Dian Fragment, which is consistent with a weak coupling between the lower crust and the upper lithospheric mantle. However, shear-wave splitting data cannot resolve the radial variations of seismic anisotropy, and therefore cannot locate unambiguously the deformation at depth. By contrast, radial and lateral variations in seismic anisotropy can be assessed by surface-wave data, which sample different depth ranges depending on their periods^26^. The mapping of isotropic and anisotropic surface-wave velocity anomalies at selected periods is based on the measurements and inversion of a collection of dispersion curves. These maps can then be linked to appropriate depth ranges through phase-velocity sensitivity kernels (Supplementary Figure S14). The presence of layers with different anisotropic patterns, as observed in various regions^27^,^28^, indicates the succession of different episodes or mechanisms of deformation.

Here, we build maps of the fundamental-mode of Rayleigh-wave phase velocity for the eastern Tibetan Plateau and the western margins of the Sino-Korean and Yangtze cratons using teleseismic data from the China National Seismic Network^29^, following a two-step method (see Supplementary Information for details). First, we used the cross-correlation approach to measure the interstation phase velocity dispersion curves^28^, resulting in a set of dispersion curves for 531 interstation paths. A special effort was made on the quality of the measurements to prevent scattered waves, multi-pathing or off-great-circle propagation from biasing our measurements. We then invert this collection of dispersion curves for maps of both isotropic and anisotropic anomalies of Rayleigh-wave phase velocity at selected periods^27^,^28^,^30^,^31^. Resolution tests are discussed in Supplementary Information.

## Results

**Figure 2:**
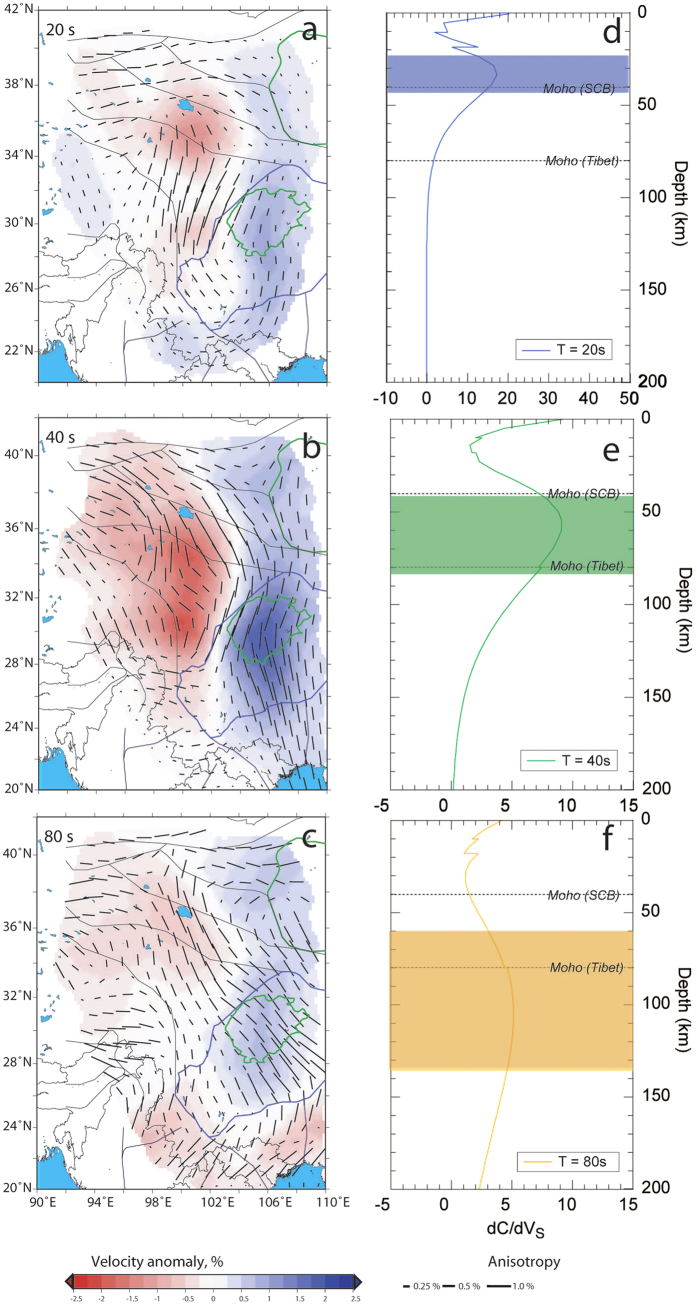
(**a**–**c**) Maps of isotropic (color code) and azimuthal anisotropic (black bars) Rayleigh-wave phase velocity at selected periods, (**a**) 20, (**b**) 40, and (**c**) 80 seconds. The direction and length of the bars denote the direction of fast propagation and amplitude of anisotropic anomalies, respectively. (**d**–**f**) Depth sensitivity kernels at (**d**) 20, (**e**) 40, and (**f**) 80 seconds, with the color-shaded area indicating the depths range of maximum sensitivity. The depth of the Moho beneath eastern Tibet and beneath the western margins of the Sino-Korean and Yangtze cratons are also indicated. The model used to calculate the sensitivities is the continental AK135 model^46^. This figure is generated using the Generic Mapping Tools^45^ and GIMP 2.6.

Our preferred models are displayed in Fig. 2 for periods of 20 s, 40 s, and 80 s. Models for other periods are shown in Supplementary Figures S15 and S16. These models show variations in Rayleigh-wave phase-velocity anomalies with both horizontal location and period.

In the period range 20–40 s, the isotropic structure (background color in Fig. 2) is dominated by an east-west dichotomy, with slow velocities in the western part of the model, and fast velocities in its eastern part. The strongest relative velocity anomalies are found at the period of 40 s, with fast anomalies of up to 2% (relative to regional average) beneath the Sichuan Basin, and slow anomalies of up to −2.5% in the eastern Songpan-Ganze block and around the eastern margin of the Qaidam Basin. North and south of the Sichuan Basin, seismic velocities are faster than average, but with a small amplitude, typically around 1%. The east-west dichotomy is still present, but much attenuated at periods of 80 s and longer. The isotropic part of the model thus reveals clear differences in the lithospheric structure of the main units of the region, in agreement with previous seismological models based on regional traveltime tomography^17^,^18^, surface-wave tomography at a local scale^11^,^19^,^20^,^21^,^22^ and surface-wave tomography at larger scales^32^,^33^,^34^.

The anisotropic pattern, represented by black bars in Fig. 2, does not substantially change in the period range 20–40 s. At these periods, the direction of fast propagation bends around the border of the Tibetan Plateau. In the northern part, the anisotropic fast direction is almost EW, and rotates towards a NS direction around 100°E. North of 34°N, the direction is NW-SE, and turns to slightly NE-SW south of this latitude. Interestingly, this change is well correlated with the contrast in isotropic velocity. In the eastern part of our model (east of 104°E longitude), covering the western borders of the Sino-Korean and Yangtze cratons, the direction of fast propagation is again NS. Note that this trend is observed up to 60 s period. At periods of 80 s and longer, the fast axis of anisotropy is mostly NW-SE throughout the area covered by our model, except in its northern part (36°N and north), which is again dominated by an EW direction of fast propagation. Interestingly, the NW-SE trend south of 36°N latitude is consistent with the regional Absolute Plate Motion (APM) determined with a fixed European plate and various reference frames^35^,^36^,^37^. Good agreement can be seen among the fast axes of polarization of the SKS measurements^13^,^24^,^25^,^38^, surface-wave tomography^21^,^22^ and the direction of fast propagation predicted by the anisotropic part of our models. In some regions, the SKS measurements seem mostly sensitive to the mantle deformation, as expected; but in some parts of our models (e.g., 34°N and 96°E), we also see a match between the crustal anisotropy and the SKS splitting results.

**Figure 3:**
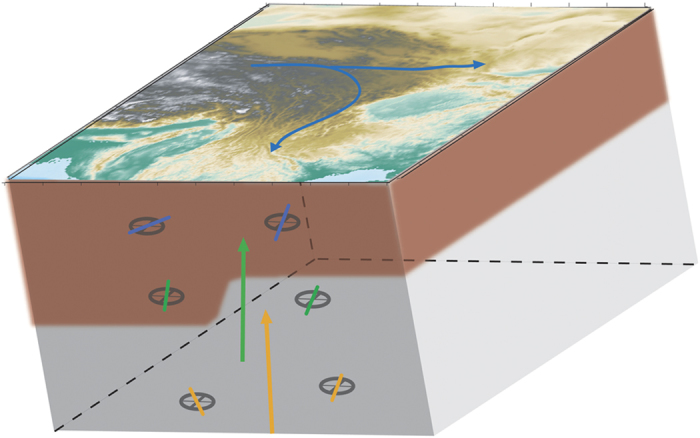
Sketch of the flow in the region, displaying the average anisotropic patterns at different periods (blue - 20 s, sampling the crust; green - 40 s, sampling around the Moho; and orange - 80 s, sampling the lithospheric mantle) and proposed flow (arrows with the same color code). This figure is generated using the Generic Mapping Tools^45^ and GIMP 2.6.

Our models have strong implications for the structure and dynamics of the lithosphere beneath Tibet. The main findings and interpretations in terms of crustal and lithospheric dynamics are summarized in Fig. 3. The most striking result in our isotropic model is the strong velocity contrast between central Tibet and the Sino-Korean and Yangtze cratons in the period range 20–40 s. We interpret this dichotomy as a change in the crustal thickness. Beneath Tibet, thickness of the crust reaches 80 km, whereas it is only 35–40 km beneath the Sichuan Basin^1^,^5^,^32^,^33^,^34^,^39^. Therefore, at 20–40 s, the Rayleigh waves sample the lithospheric mantle in Sino-Korean and Yangzte cratons, whereas they still sample the crust, which is seismically slower, beneath Tibet. Because we use an average regional model for each period, it is difficult to interpret the local variations of the velocity perturbations in terms of absolute velocities. However, the amplitudes of the anomalies (±2.5%) are consistent with the velocity contrast measured by surface-wave tomography at similar depths^32^,^33^. In the period range of 20–40 s, the fast velocity anomalies observed north of 28°N may be related to the Central Asian Orogenic Belt, i.e. the boundary between the Tibetan Plateau and the Tarim and North China cratons^40^,^41^.

The anisotropic pattern in our model provides important clues on the dynamics of the Tibetan Plateau. Based on GPS, geologic, and shear-wave splitting data, it has been argued that the lower crust and the lithospheric mantle in this region are coupled^15^. Modeling of quasi-Love wave^11^ found evidence for vertically coherent deformation in Tibet. Other studies^9^,^10^,^42^ arrived at the opposite conclusion, proposing that the lower crust is animated by a ductile flow. Because the anisotropic pattern we observe at the periods of 80 s and longer, which, in this area, sample the lithospheric mantle, strongly differs from that in the period range 20–40 s (sampling the lower crust), our model strongly supports a mechanical decoupling. Following the decoupling hypothesis, crustal material extruded from Tibet flows clockwise around the Eastern Himalayan Syntaxis into southeastern Tibet and Yunnan Province, and across the Red River Shear Zone. Again, this is consistent with the rotation of the anisotropy observed in our models (in the period range 20–40 s), and the drastic change in anisotropy along the Red River Shear Zone. By contrast, in the western margins of the Sino-Korean and Yangzte cratons, where the Moho is shallower (35–40 km), the anisotropic pattern remains unchanged throughout the period range 20–60 s, suggesting that in this region, the crust and lithospheric mantle are mechanically coupled, and experience a NS flow (Fig. 2). The anisotropic anomalies we observe give further constraints on the continuity of the crustal flow along the eastern boundary of Tibet. Using GPS measurements, a gap is found in the flow at the latitude of the Sichuan Basin^4^. Our results, however, display a continuous flow with a slight change of direction beneath central Tibet (Fig. 2), in agreement with the dynamic stress model^43^, geodetic and seismic data^12^, geological observations^7^ and magnetotelluric imaging^8^. The change in the direction (a slight clockwise rotation) of the fast axis may be linked to the Sichuan Basin, known to be the remnant of a cratonic, stiff, core^9^. Due to lower temperatures and chemical differences, this region may be stiffer than the surrounding crust, thus diverting the crustal flow. It is interesting to note that anisotropic maps at periods sampling the Tibetan crust (20–40 s) are consistent with SKS-splitting studies^13^,^24^,^25^,^38^, suggesting that in this region, the SKS waves are mostly sensitive to the middle and lower crust. At latitudes of 38°N and higher, the anisotropy is roughly EW. In the central part of our model (roughly 28°N and 100°E), the anisotropy is NS. North of the Sichuan Basin, EW fast axis of anisotropy is found, whereas it rotates to NS west of the Sichuan Basin. This is in agreement with the previous SKS-splitting measurements in the region^13^,^24^,^25^,^38^. At periods of 80 s and longer, the anisotropic pattern is, on the other hand, consistent with the Absolute Plate Motion^35^,^36^,^37^ for this region.

## Conclusion

Our Rayleigh-wave phase-velocity model highlights important structural changes and dynamic processes in the eastern Tibetan lithosphere and its surroundings. Isotropic anomalies map the main units of this region, including the Yangtze and Sino-Korean cratons and the Tibetan crust, and clearly identify the boundary between these units, which is associated with a step in the Moho depth. Lateral and vertical patterns of anisotropy provide more details on the tectonic evolution of the region. Our anisotropic maps resolve two different anisotropic layers beneath eastern Tibet, supporting a mechanical decoupling between the crust and lithospheric mantle in this region. By contrast, the vertical continuity of the anisotropic pattern beneath the Yangtze and Sino-Korean cratons indicates that the crust and lithospheric mantle are mechanically coupled in these cratons. The mechanical coupling or decoupling may be controlled by regional tectonic stress, rock properties and physical conditions (such as temperature) which influence the rheology of the lithosphere. Additional modeling, in particular geodynamical numerical experiments, should help to clarify this point.

## Methods

To build anisotropic maps of the fundamental-mode Rayleigh-wave phase velocity beneath eastern Tibet, we followed a two-step method, which consists in first measuring a collection of dispersion curves between pairs of stations, and then inverting the collection of curves for maps of isotropic and anisotropic phase-velocity anomalies at selected periods.

The dataset is a collection of selected broadband waveforms recorded at 32 seismic stations from the China National Seismic Network^29^. The stations are fairly evenly distributed in our study region, thus providing more or less even sampling of the region. The two-station method, which we employ to measure the dispersion curves between two stations, requires that the angle between the great circles connecting a given pair of stations and that connecting this pair and the earthquake epicenter are not too large. In this study, we set an upper limit of 10° for this angle. Epicentral distances are between 2° and 170°, and all interstation distances are in the range of 250–2,500 km. Following these criteria, we extracted 46,490 surface-wave records from 467 events.

For each of the 531 station pairs, we measured a phase-velocity dispersion curve for the fundamental Rayleigh mode using the cross-correlation approach of the two-station technique. This approach allows for the measurement of dispersion curves in a broad period range, in our case, between 10 and 200 s. For each selected event, the vertical-component displacements recorded at the two stations of each pair are cross-correlated. To minimize the effects of noise and interferences, the cross-correlation function is first filtered with a frequency-dependent Gaussian band-pass filter. The cross-correlation is then transferred into the frequency domain, and its complex phase is used to calculate the phase velocity. All dispersion curve fragments for a specific station pair are then assembled and averaged to construct a single dispersion curve between the two stations, and outliers are discarded.

After deriving the dispersion curves for the 531 interstation paths, we invert them for both isotropic and anisotropic (2*ψ* and 4*ψ*) Rayleigh-wave phase-velocity maps at selected periods. At each point of the model, the total velocity anomaly can be parameterized with 5 coefficients: one for the isotropic phase-velocity variation, *δC*_*iso*_, 2 for the 2*ψ*-anomaly, *A*_2*ψ*_ and *B*_2*ψ*_, and 2 for the 4*ψ*-anomaly, *A*_4*ψ*_ and *B*_4*ψ*_:


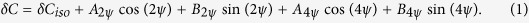


In our models, the amplitude of the 2*ψ* anisotropy is larger than that of the 4*ψ* anisotropy by a factor 2 to 5 (see Supplementary Information Figure S8). Furthermore, inverting for the 4*ψ* terms does not alter the patterns of isotropic and 2*ψ* anisotropy anomalies (left and middle columns in Figure S9). For information, the right column in Figure S9 plots the 4*ψ* anisotropy inferred from our preferred models. It is worth noting that previous studies using the same method^27^ found that the 4*ψ* terms, while having an amplitude comparable to that of the 2*ψ* terms, do not significantly improve the *χ*^2^, and are therefore not needed to explain the data.

Geographically, the model is parameterized on a triangular grid of knots^44^, with a grid spacing of 80 km. Due to variable data coverage depending on the period, the size of the grid is different at each period. Good coverage (which is obtained for periods longer than 30 s) allows for small grid spacing leading to a grid of 425 knots at 80 s. When coverage is poor (for periods shorter than 30 s), the grid covers a reduced area (e.g., 306 knots at 20 s). Each dispersion curve yields the average phase velocity along the path linking the two stations as a function of period, and the total average velocity anomaly along this path may be written as the integral of local anomalies at each grid knot sampled by the given path,


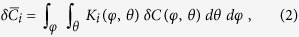


where the local anomalies *δC*(*φ*, *θ*) are weighted with respect to the sensitivity kernels *K*_*i*_(*φ*, *θ*). The kernel provides the contribution at each knot on a specific path to the total velocity anomaly.

Waveform data for this study are provided by the Data Management Centre of China National Seismic Network at Institute of Geophysics, China Earthquake Administration (SEISDMC, doi:10.7914/SN/CB)^29^. Figures were generated with the Generic Mapping Tools^45^.

## Additional Information

**How to cite this article**: Legendre, C. P. *et al.* Rayleigh-wave dispersion reveals crust-mantle decoupling beneath eastern Tibet. *Sci. Rep.* **5**, 16644; doi: 10.1038/srep16644 (2015).

## Supplementary Material

Supplementary Information

## Figures and Tables

**Figure 1:**
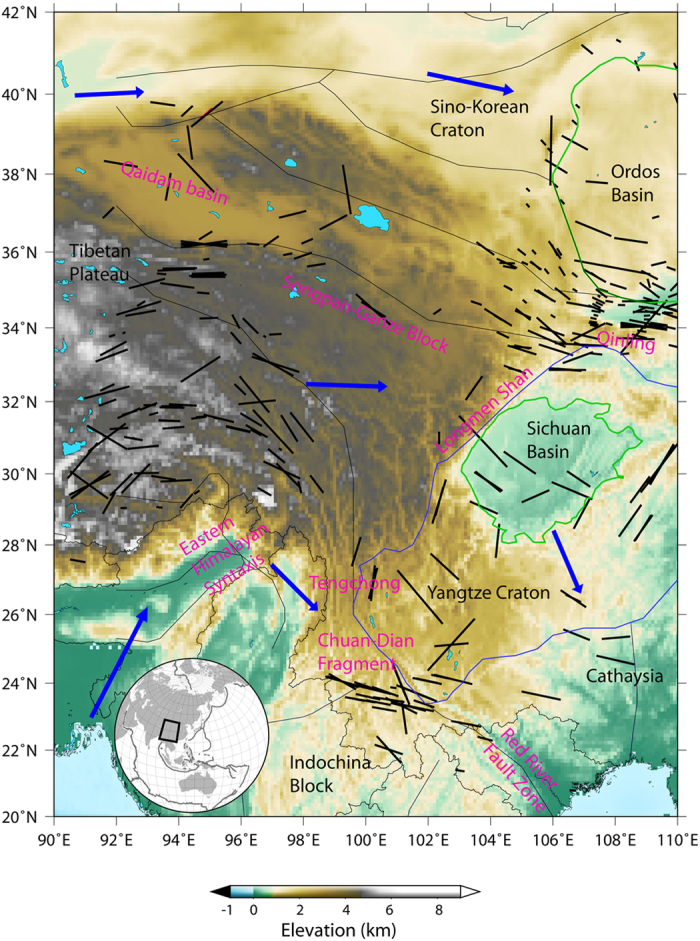
Simplified tectonic map of eastern Tibet. Names of tectonic units are highlighted in black (Sino-Korean and Yangtze Cratons, Ordos and Sichuan Basins, Tibetan Plateau, Cathaysia and Indochina Blocks) and important tectonic features are highlighted in purple (Chuan-Dian Fragment, Eastern Himalayan Syntaxis, Longmen Shan, Qaidam Basin, Qinling, Red River Fault Zone, Songpan-Ganze Block and Tenchong). Thick black lines show the SKS-splitting measurements. Blue arrows represent the absolute plate motion in the region. The background color indicates the topographic relief. This figure is generated using the Generic Mapping Tools^45^ and GIMP 2.6.
