# Supplementary material for: Rayleigh-wave dispersion reveals crust-mantle decoupling beneath eastern Tibet
Source: Sci Rep. 2015 Nov 9;5:16644. doi: 10.1038/srep16644 (PMC4637932; doi:10.1038/srep16644)
Supplement: Supplementary Information [file srep16644-s1.pdf]

Rayleigh-wave dispersion reveals crust-mantle decoupling beneath eastern Tibet – Supplementary Information

Cédric P. Legendre[1,2,+,*], **Frédéric Deschamps**[1,+], **Li Zhao**[1,+], **and Qi-Fu Chen**[3,+]

[1]Institute of Earth Sciences, Academia Sinica, 128 Academia Road, Sec. 2, Nangang, Taipei 11529, Taiwan
[2]now at Department of Geosciences, National Taiwan University, No. 1, Sec 4, Roosevelt Rd., Taipei 10617, Taiwan
[3]Key Laboratory of Earth and Planetary Physics, Institute of Geology and Geophysics, Chinese Academy of Sciences, Beijing 100029, China.
[*]legendre@earth.sinica.edu.tw
[+]these authors contributed equally to this work

ABSTRACT

Supplementary Information

Seismic data and inversion.

Data subsection.

We build anisotropic maps of the fundamental-mode Rayleigh-wave phase velocity beneath eastern Tibet (covering 20°N - 42°N, and 90°E - 110°E) following a two-step method, which consists in first measuring the dispersion between many station pairs, and then inverting the collection of dispersion curves for maps of isotropic and anisotropic phase-velocity anomalies at selected periods.

The data we use are broadband waveforms recorded at 32 seismic stations from the China National Seismic Network.[1] The stations are fairly evenly distributed in our study region (Figure S1), which provide more or less even sampling of the region. The two-station method, which we employ to measure the dispersion curves between two stations, requires that the angle between the great circles connecting a given pair of stations and that connecting this station pair and the earthquake epicenter are not too large. In this study, we set an upper limit of 10° for this angle. Epicentral distances are between 2° and 170°, and all interstation distances are in the range of 250-2,500 km. Following these criteria, we extracted 46,490 surface-wave records from 467 events (Figure S2).

Dispersion curves

For each of the 531 station pairs, a phase-velocity dispersion curve can be measured for the fundamental Rayleigh mode using the two-station technique.[2,3] Here, we used the cross-correlation approach,[4] which allows for the measurement of dispersion curves in a broad period range (in our case, between 10 and 200 s). This technique has been widely used and further developed in the last decade.[5–18]

For each selected event, the vertical-component displacements recorded at the two stations of each pair are cross-correlated (Figure S3). To minimize the effects of noise and interferences, the cross-correlation function is first filtered with a frequency-dependent Gaussian band-pass filter. Side lobes caused by correlations of the fundamental mode with scattered waves and higher modes are then down weighted by the application in the time domain of a frequency-dependent Gaussian window to the filtered cross-correlation function. This approach to filtering and windowing is effective as long as the fundamental mode has the largest amplitude in the seismogram, which is always true at epicentral distances greater than 1,200 km, and therefore dominates the contribution to the cross-correlation function (Figure S3). The cross-correlation is then transferred into the frequency domain, and its complex phase is used to calculate the phase-velocity.

Recent studies[19,20] showed that some paths may not be close to the great circle. While calculating the dispersion curves, strong off great circle propagation may result in biases in phase-velocity measurements. We have however identified such potential errors at the early stage of our measurements and removed them when building the dispersion curves. Because we have been looking at every seismogram independently, we could select (or reject) specific parts of the dispersion curve for each event (Figure S4-top). Sometimes, as shown on Figure S4-bottom, raw dispersion curves show strong deviations from the refernce curve (bumps or inconsistent low or high velocities in the curve). The bumps (indicated by the orange circles) may

originate from various causes, such as errors in the data, noise, or correlations of the fundamental mode with scattered waves and higher modes. For the part of the dispersion curve marked by the green circle, both phase-velocity and the first derivative are different at 5.10^{-2}Hz compared to the segment between 3.10^{-2}Hz and 4.10^{-2}Hz. This can be caused by noise, errors in the data or even propagation effects (off-great circle for example). It is fairly easy to spot those, as they display different velocity values and/or different first derivatives. Those sections of the dispersion curves are rejected directly. Those deviations could originate from interactions between fundamental and higher mode wavetrains, or off-great-circle propagation, as noted above, but in all cases we did not take those into account.

Generally speaking, seismic-wave diffraction and the interference of fundamental Rayleigh wave with higher modes can bias the measurements. These effects, however, have a strong frequency dependence, which manifests itself in the irregularities and roughness in the measured curves. Therefore, only the smooth portions of the curve are retained (red portion in Figure S4). In the next step, All dispersion curve fragments for a specific station pair measured for all suitable events are then merged together and averaged (Figure S5), and outliers are discarded to derive a single dispersion curve between the two stations (Figure S6).

The 531 dispersion curves we measured (Figure S6) do not cover the entire period range (10-200 s) evenly. As a result, the path coverage changes strongly with period (Figure S7), leading to variations in the resolution of our model as a function of period. For example, at short periods (shorter than 30 s) fewer dispersion curves are available to provide information. By contrast, almost all the dispersion curves we obtained sample periods of 30 s and longer, leading to a very good coverage at these periods, as indicated by the azimuthal coverage for specific periods (Figure S7).

Inversion scheme

After deriving the dispersion curves for the 531 interstation paths, we invert them for both isotropic and anisotropic (2ψ and 4ψ) Rayleigh-wave phase-velocity maps at selected periods. At each point of the model, the total velocity anomaly can be parameterized with 5 parameters: one for the isotropic phase-velocity variation, δC_{iso}, 2 for the 2ψ-anomaly, $A_{2\psi}$ and $B_{2\psi}$, and 2 for the 4ψ-anomaly, $A_{4\psi}$, and $B_{4\psi}$. The total anomaly is then:

$$\delta C = \delta C_{iso} + A_{2\psi} cos(2\psi) + B_{2\psi} sin(2\psi) + A_{4\psi} cos(4\psi) + B_{4\psi} sin(4\psi). \qquad (1)$$

The amplitudes of azimuthal velocity variation (Λ) and the directions of fast propagation (Θ) of the 2ψ- and 4ψ-anisotropy are then given by:

$$\begin{cases} \Lambda_{2\psi} = \sqrt{A_{2\psi}^2 + B_{2\psi}^2} \\ \Theta_{2\psi} = \frac{1}{2}\, arctan(\frac{B_{2\psi}}{A_{2\psi}}) \end{cases} \quad and \quad \begin{cases} \Lambda_{4\psi} = \sqrt{A_{4\psi}^2 + B_{4\psi}^2} \\ \Theta_{4\psi} = \frac{1}{4}\, arctan(\frac{B_{4\psi}}{A_{4\psi}}) \end{cases} \quad . \qquad (2)$$

Previous studies[10,13,21–24] have pointed out that the contribution of 4ψ terms may be non-negligible, which is why we have included them in our inversions. In our models, we found that the amplitude of the 2ψ anisotropy is larger than that of the 4ψ term by a factor 2 to 5 (Figure S8). More importantly, we performed inversions with isotropic and 2ψ terms only, and found that adding 4ψ terms, as we did in our models, does not alter the patterns and amplitude of isotropic and 2ψ anisotropy anomalies (Figure S9). The left column in Figure S9 show the distribution of 4ψ anisotropy obtained for our preferred models. Previous studies using the same method showed that although the 4ψ terms may have amplitudes comparable to those of the 2ψ terms, they are not needed to explain the raw data,[10,13] i.e. they do not provide significant reduction in the reduced χ^2 compared to inversions in which they are omitted. Therefore, when discussing the results of our inversions, we will not attempt to interpret the 4ψ terms and focus only on the isotropic and 2ψ anisotropic anomalies.

Geographically, the model is parameterized on a triangular grid of knots[25] with a grid spacing of 80 km. Due to variable data coverage depending on the period, the size of the grid is different at each period. Good coverage (which is obtained for periods of 30 s and longer) allows for small grid spacing leading to a grid of 425 knots at 80 s. When coverage is poor (for periods shorter than 30 s), the grid covers a reduced area (e.g., 306 knots at 20 s). Each dispersion curve yields the average phase velocity along the path linking the two stations as a function of period, and the total average velocity anomaly along the path may be written as the integral of local anomalies at each grid knot sampled by the given path,

$$\delta \bar{C_i} = \int_{\varphi}\int_{\theta} K_i(\varphi,\theta)\; \delta C(\varphi,\theta)\; d\theta\; d\varphi\ , \qquad (3)$$

where the local anomalies $\delta C(\varphi,\theta)$ are weighted with respect to the sensitivity kernels $K_i(\varphi,\theta)$. The kernel provides the contribution at each knot on a specific path to the total phase-velocity anomaly.[26]

Tests on regularization parameters and resolution

Regularization parameters

In this section, we discuss the influence of regularization parameters in the inversion such as norm damping and smoothing on the resulting model.

Resolution is mainly controlled by the cumulative sensitivity of the available data to isotropic and anisotropic perturbations of the medium, but also by additional *a priori* constraints such as norm damping and smoothing. The norm damping is used to constrain the amplitudes of phase-velocity perturbations during the inversion. Therefore, investigating the influence of this parameter allows us to calibrate our inversion.

As shown in Figure S10(a-f), the isotropic damping does not affect the lateral distribution of the anomalies or the anisotropic part. Increasing norm damping decreases the amplitude of the velocity anomaly. Therefore this parameter has been set to be consistent with the initial dispersion curves, from which the maximum amplitude of the anomalies were estimated.

Lateral smoothing penalizes the difference between the anomaly at a knot and the average of the anomalies at its neighboring knots by minimizing the first and second spatial derivatives of velocity anomalies. The lateral smoothing weight is set independently for each period. We apply a stronger smoothing with decreasing path coverage. Figure S11 shows the effect of smoothing on the resulting velocity model at a period of 50 s. If the smoothing weight is too low (Figure S11-a), small-scale artifacts may appear in the regions with poor data coverage (i.e. at the borders of the model). If the smoothing weight is instead too high, information on small-scale features is lost (Figure S11-f). Therefore, we imposed a smoothing weight large enough in order to avoid artifacts yet still allowing the resolution of small-scale anomalies. Figure S11(a-f) further shows that the anisotropic pattern remains mostly unchanged, indicating the robustness of the inversion.

Similar tests have been conducted for the anisotropic part (Figure S12). When the anisotropic smoothing factor increases, local changes of fast direction of the anisotropy are weakened, and only the average direction of the anisotropy in the region (for a selected period) is obtained.

Resolution tests

To test the robustness of the structures imaged by our inversion, we performed a series of resolution tests (Figure S13). For each test, an input test model is first created with representative patterns for both isotropic and anisotropic anomalies (Figure S13, top row). We then generate synthetic data from these input models, and invert these synthetic data under the same conditions as the real data (Figure S13, bottom row). In particular, the same amount of smoothing and damping, and the same path coverage are used for a given period. This allows testing for the reconstruction of both isotropic anomalies and anisotropic patterns. The retrieval of the isotropic anomalies should display correct amplitudes and locations. The anisotropic features should be retrieved with consistent directions and amplitudes.

Distortions in the output models may result from several sources, but mainly a lack of lateral resolution and smearing between isotropic and anisotropic terms. To evaluate the smearing between isotropic and anisotropic terms, we define synthetic models in which isotropic and anisotropic anomalies are either constant throughout the region, or set to zero (Figure S13). The retrieved models indicate that the smearing between isotropic and anisotropic terms is small. In particular, the isotropic patterns are retrieved for all periods with small lateral variations. In regions with denser data coverage (between 40 and 100 s) some artifacts appear in the isotropic part. Note that these artifacts mostly occur at the peripheral knots, where the coverage is poorer. These regions should thus be interpreted with caution, due to the possible small-scale artifacts and perturbations induced by the lack of path coverage. Similarly, the input anisotropic structure is generally properly retrieved. Results of these tests show only small discrepancies between the input and output anisotropic anomalies both in the azimuth of fast direction and in amplitude. Furthermore, the output model did not show substantial isotropic perturbations, indicating very little leakage between isotropic and anisotropic terms. Note that the resolution tests presented here addressed the coverage by the data only and no additional noise has been added. The main sources of errors in the actual inversions are the locations and origin times of the events, errors in the dispersion curves and diffraction effects, which are not accounted for by the approximate sensitivity kernels ($K_i(\varphi, \theta)$ in eq. 3) that we used. Given the very substantial redundancy of our coverage provided by the hundreds of crossing paths, random errors are unlikely to have any systematic impact on the results.[27]

The resulting maps can then be associated with appropriate depth ranges through phase-velocity sensitivity kernels at specific periods (Figure S14). Final models for all periods are displayed in Figure 2 and Figures S15 and S16.

References

1. Zheng, X.-F., Yao, Z.-X., Liang, J.-H. & Zheng, J. The role played and opportunities provided by IGP DMC of China National Seismic Network in Wenchuan earthquake disaster relief and researches, *Bull. Seismol. Soc. Am.*, **100**, 2866–2872 (2010).

2. Sato, Y. Analysis of dispersed surface waves by means of Fourier transform I., *Bull. Earthquake Res. Tokyo Univ*, **33**, 33–48 (1955).

3. Knopoff, L. Observation and inversion of surface-wave dispersion, *Tectonophysics*, **13**, 497–519 (1972).

4. Meier, T., Dietrich, K., Stöckhert, B. & Harjes, H.-P. One-dimensional models of shear wave velocity for the eastern Mediterranean obtained from the inversion of Rayleigh wave phase velocities and tectonic implications, *Geophys. J. Int.*, **156**, 45–58 (2004).

5. Endrun, B., Meier, T., Bischoff, M. & Harjes, H.-P. Lithospheric structure in the area of Crete constrained by Receiver Functions and dispersion analysis of Rayleigh phase velocities, *Geophys. J. Int.*, **158**, 592–608 (2004).

6. Lebedev, S., Meier, T. & van der Hilst, R. Asthenospheric flow and origin of volcanism in the Baikal rift area, *Earth Planet. Sci. Lett.*, **249**, 415–424 (2006).

7. Zhang, X., Paulssen, H., Lebedev, S. & Meier, T. Surface wave tomography of the Gulf of California, *Geophys. Res. Lett.*, **34** (2007).

8. Adam, J. M.-C. & Lebedev, S. Azimuthal anisotropy beneath Southern Africa from very broad-band surface-wave dispersion measurements, *Geophys. J. Int.*, **191**, 155–174 (2012).

9. Darbyshire, F. A. & Eaton, D. W. The lithospheric root beneath Hudson Bay, Canada from Rayleigh wave dispersion: No clear seismological distinction between Archean and Proterozoic mantle, *Lithos*, **120**, 144–159 (2010).

10. Deschamps, F., Lebedev, S., Meier, T. & Trampert, J. Azimuthal anisotropy of Rayleigh-wave phase velocities in the east-central United States, *Geophys. J. Int.*, **173**, 827–843 (2008).

11. Endrun, B. *et al.* S velocity structure and radial anisotropy in the Aegean region from surface wave dispersion, *Geophys. J. Int.*, **174**, 593–616 (2008).

12. Erduran, M., Endrun, B. & Meier, T. Continental vs. oceanic lithosphere beneath the eastern mediterranean sea–implications from Rayleigh wave dispersion measurements, *Tectonophysics*, **457**, 42–52 (2008).

13. Fry, B., Deschamps, F., Kissling, E., Stehly, L. & Giardini, D. Layered azimuthal anisotropy of Rayleigh wave phase velocities in the European Alpine lithosphere inferred from ambient noise, *Earth Planet. Sci. Lett.*, **297**, 95–102 (2010).

14. Endrun, B., Lebedev, S., Meier, T., Tirel, C. & Friederich, W. Complex layered deformation within the Aegean Crust and Mantle revealed by seismic anisotropy, *Nat. Geo.*, **4**, 203–207 (2011).

15. Roux, E. *et al.* Joint inversion of long-period magnetotelluric data and surface-wave dispersion curves for anisotropic structure: Application to data from central Germany, *Geophys. Res. Lett.*, **38**, 5pp (2011).

16. Legendre, C. P., Deschamps, F., Zhao, L., Lebedev, S. & Chen, Q.-F. Anisotropic Rayleigh wave phase velocity maps of eastern China, *J. Geophys. Res.*, **119**, 4802–4820 (2014).

17. Legendre, C., Chen, Q.-F. & Zhao, L. Lithospheric structure beneath the East China Sea revealed by Rayleigh-wave phase velocities, *J. of Asian Earth Sci.*, **96**, 213 – 225 (2014).

18. Legendre, C. P., Zhao, L., Huang, W.-G. & Huang, B.-S. Anisotropic Rayleigh-wave phase velocities beneath northern Vietnam, *Earth, Planets and Space*, **67**, 28 (2015).

19. Woodhouse, J. & Wong, Y. Amplitude, phase and path anomalies of Mantle waves, *Geophys. J. Int.*, **87**, 753–773 (1986).

20. Cotte, N., Pedersen, H. A., Campillo, M., Farra, V. & Cansi, Y. Off-great-circle propagation of intermediate-period surface waves observed on a dense array in the French Alps, *Geophys. J. Int.*, **142**, 825–840 (2000).

21. Montagner, J.-P. & Tanimoto, T. Global Upper Mantle tomography of seismic velocities and anisotropies, *J. Geophys. Res.*, **962**, 20337–20351 (1991).

22. Trampert, J. & Woodhouse, J. H. Global anisotropic phase velocity maps for fundamental mode surface waves between 40 and 150 s, *Geophys. J. Int.*, **154**, 154–165 (2003).

23. Beucler, É. & Montagner, J.-P. Computation of large anisotropic seismic heterogeneities (CLASH), *Geophys. J. Int.*, **165**, 447–468 (2006).

24. Polat, G., Lebedev, S., Readman, P., O'Reilly, B. & Hauser, F. Anisotropic Rayleigh-wave tomography of Ireland's crust: Implications for crustal accretion and evolution within the Caledonian Orogen, *Geophys. Res. Lett.*, **39**, 5pp (2012).

25. Wang, Z. & Dahlen, F. A. Spherical-spline parameterization of three-dimensional Earth models, *Geophys. Res. Lett.*, **22**, 3099–3102 (1995).

26. Lebedev, S. & Van Der Hilst, R. D. Global Upper-Mantle tomography with the Automated Multimode Inversion of surface and S-wave forms, *Geophys. J. Int.*, **173**, 505–518 (2008).

27. Legendre, C. P., Meier, T., Lebedev, S., Friederich, W. & Viereck-Götte, L. A shear wave velocity model of the European Upper Mantle from automated inversion of seismic shear and surface waveforms, *Geophys. J. Int.*, **191**, 282–304 (2012).

28. Kennett, B., Engdahl, E. & Buland, R. Constraints on seismic velocities in the Earth from traveltimes, *Geophys. J. Int.*, **122**, 108–124 (1995).

29. Wessel, P. & Smith, W. H. New version of the Generic Mapping Tools, *Eos Trans. AGU*, **76**, 329 (1995).

Acknowledgements (not compulsory)

The authors thank S. Lebedev who provided insight and expertise that greatly assisted this research, and Y.-C. Wang and C.-J. Chou for assistance with the measurements of the dispersion curves. This work was funded by an Academia Sinica Career Development Award (AS-098-CDA-M02), the National Science Council of Taiwan under grants NSC-101-2116-M-001-035 and NSC-101-2116-M-001-001-MY3 and by Ministry of Science and Technology (MOST) of Taiwan under grants MOST 103-2116-M-001-026-MY3.

Waveform data for this study are provided by the Data Management Centre of China National Seismic Network at Institute of Geophysics, China Earthquake Administration (SEISDMC, doi:10.7914/SN/CB).[1] Figures were generated with the Generic Mapping Tools.[29]

Additional information

The authors declare no competing financial interests.

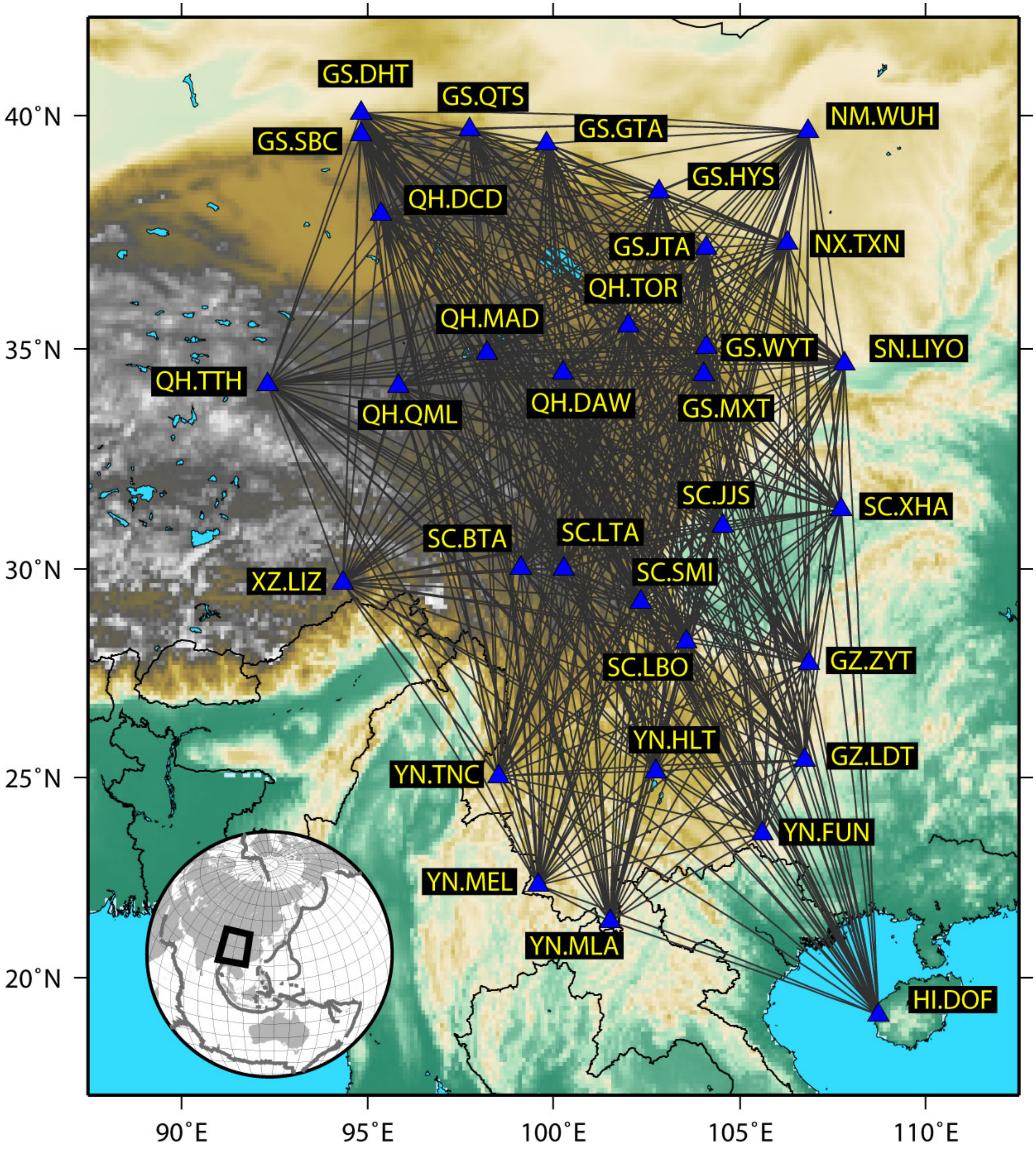

Figure S1. Stations (blue triangles) used in this study. Thin black lines linking station pairs are possible paths used to constrain the model. The box in the inset map shows the region under study. This figure is generated using the Generic Mapping Tools[29] and GIMP 2.6.

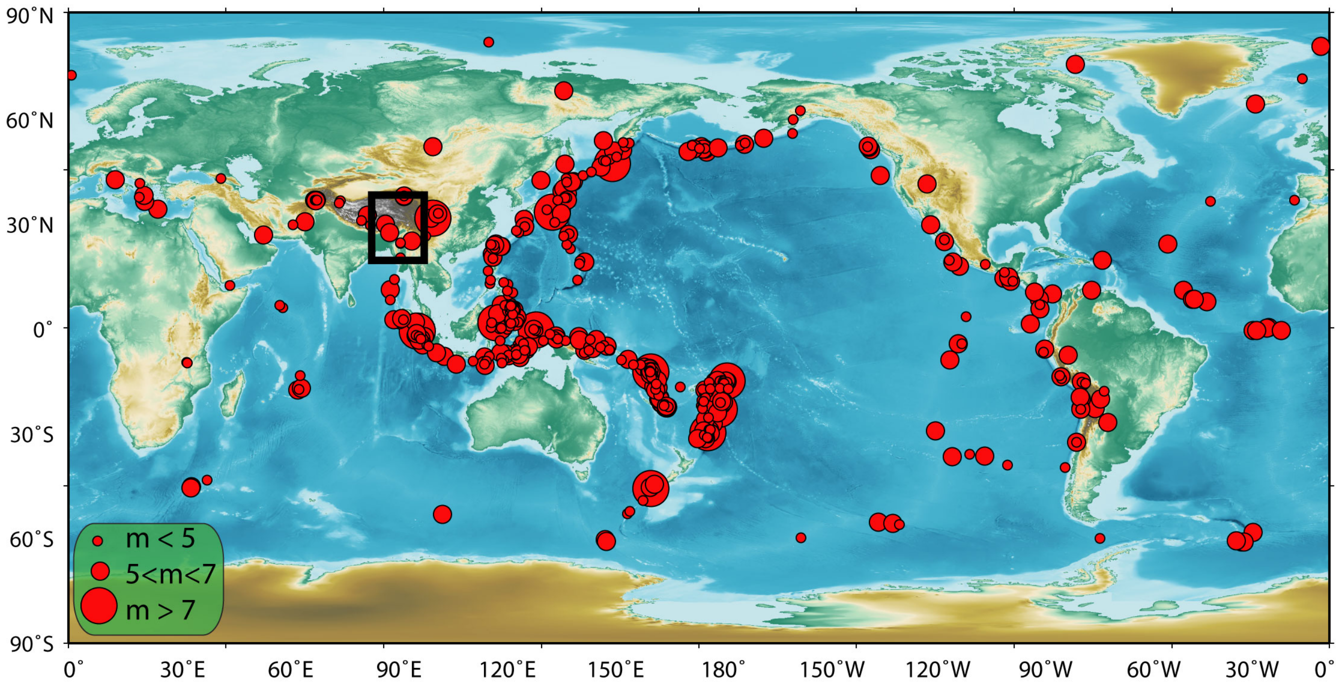

Figure S2. Events (red circles) used in this study. The magnitude of each event is indicated by the size of the circle. The black box shows the study region. This figure is generated using the Generic Mapping Tools[29] and GIMP 2.6.

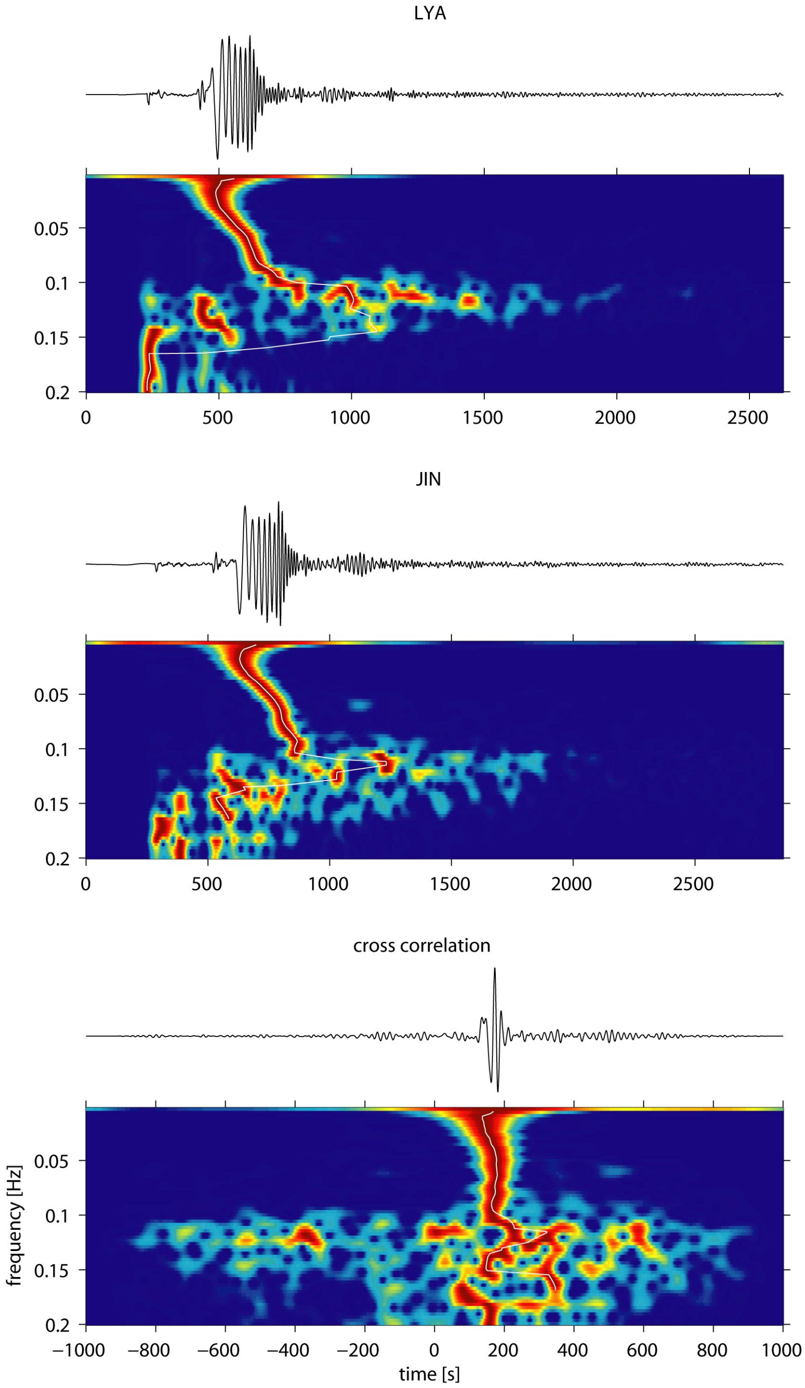

Figure S3. Vertical-component displacements recorded at two stations (top and centre). The bottom panel shows the cross-correlation between the two seismograms. The color images shows the corresponding spectro-temporal diagrams. The color indicates the amount of energy (normalized for each frequency). The white curves in the images represent the maximum energy recorded for all frequencies. This figure is generated using Matlab (2013b).

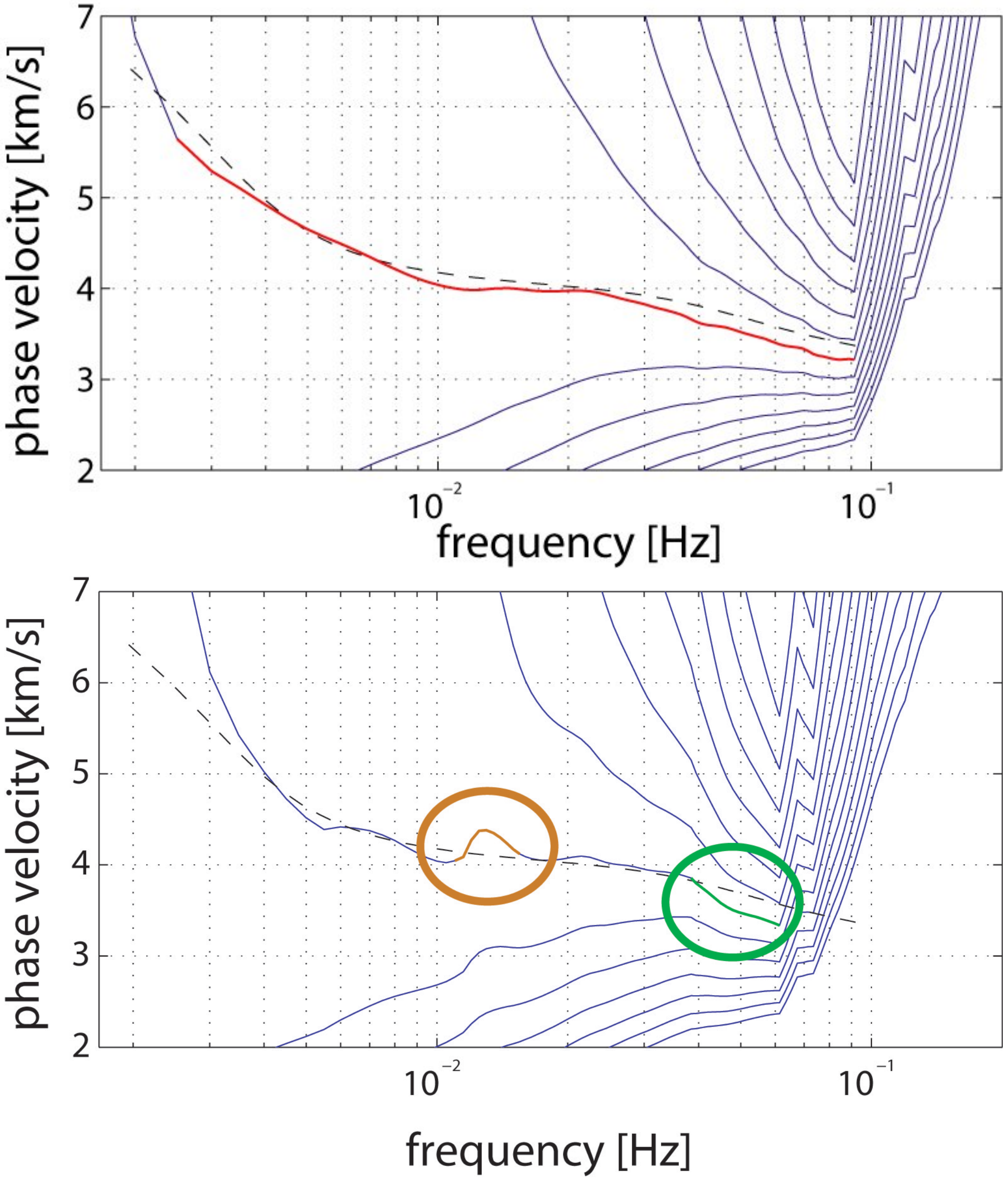

Figure S4. (Top) - Measurements of an individual Rayleigh-wave dispersion curve obtained for a specific station pair from a single event. The selected dispersion curve (solid red line) is chosen as close as possible from the predicted dispersion curve calculated from a reference model (dashed black line) amongst possible dispersion curves from the inversion (solid blue lines). (Bottom) - Some parts of the dispersion curve show bumps (orange circle) or strong deviations (green circle) to the predicted dispersion curve (dashed black line). This figure is generated using Matlab (2013b).

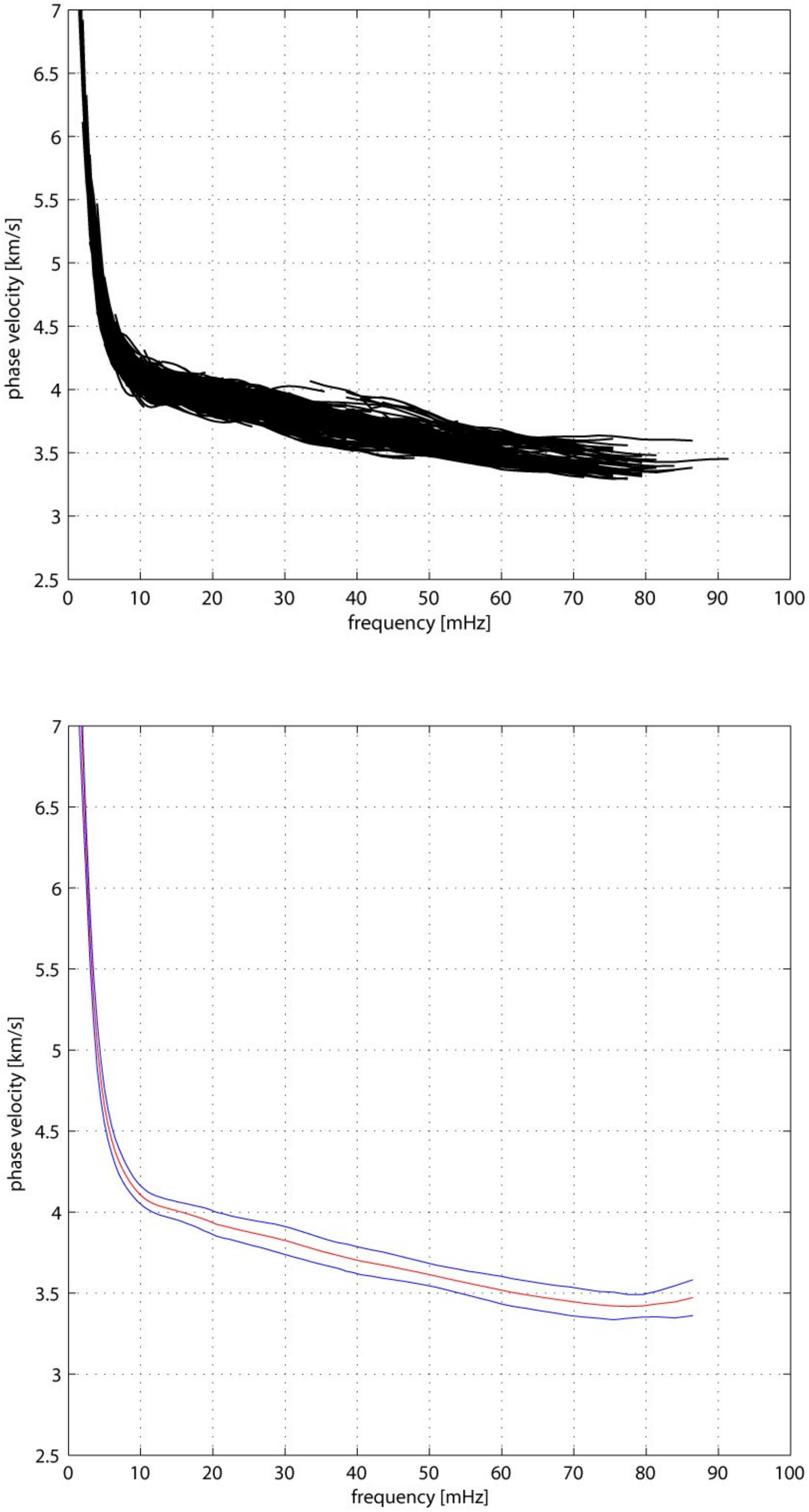

Figure S5. Individual dispersion curves measured for a specific station pair from all events (top), and the resulting averaged dispersion curve (bottom). Blue curves are for one standard deviation. This figure is generated using Matlab (2013b).

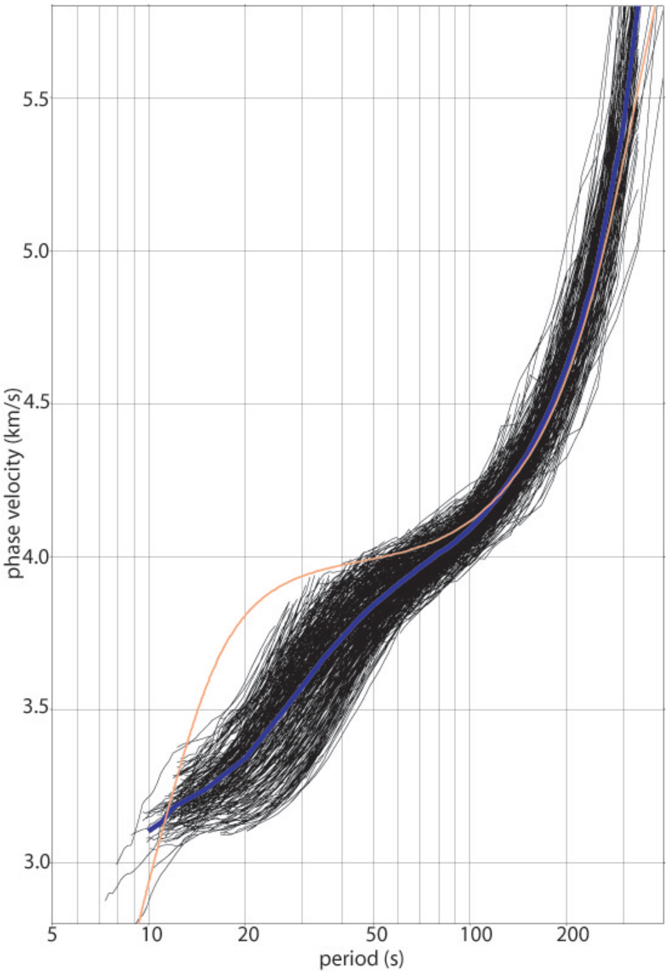

Figure S6. All measured phase-velocity curves used in this study (black). The orange curve shows the prediction by the PREM model. The average regional dispersion curve is ploted in blue. It is used as reference velocity to calculate velocity anomalies. This figure is generated using the Generic Mapping Tools[29] and GIMP 2.6.

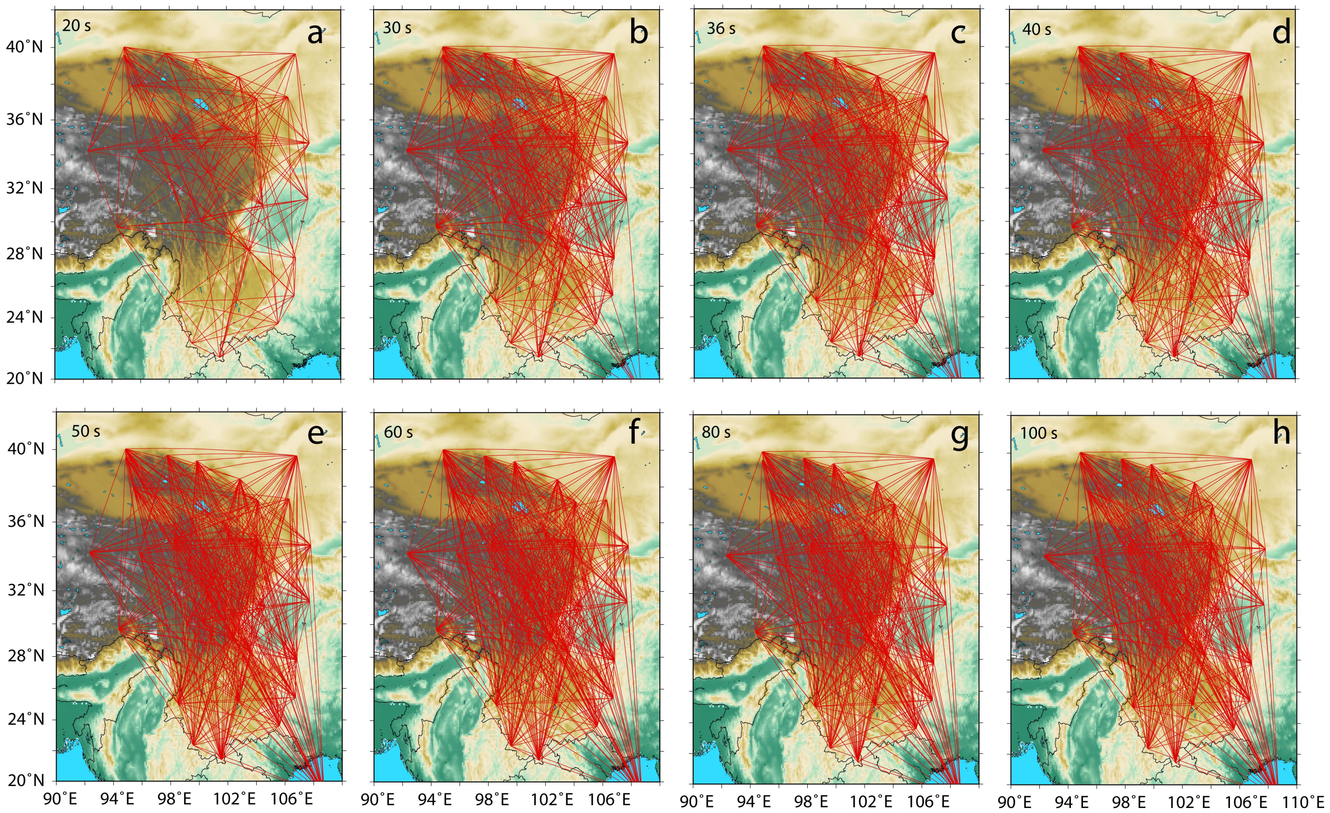

Figure S7. Interstation paths used to constrain surface-wave phase-velocity maps at specific periods. From (a) to (h) 20, 30, 36, 40, 50, 60, 80 and 100 seconds, respectively. This figure is generated using the Generic Mapping Tools[29] and GIMP 2.6.

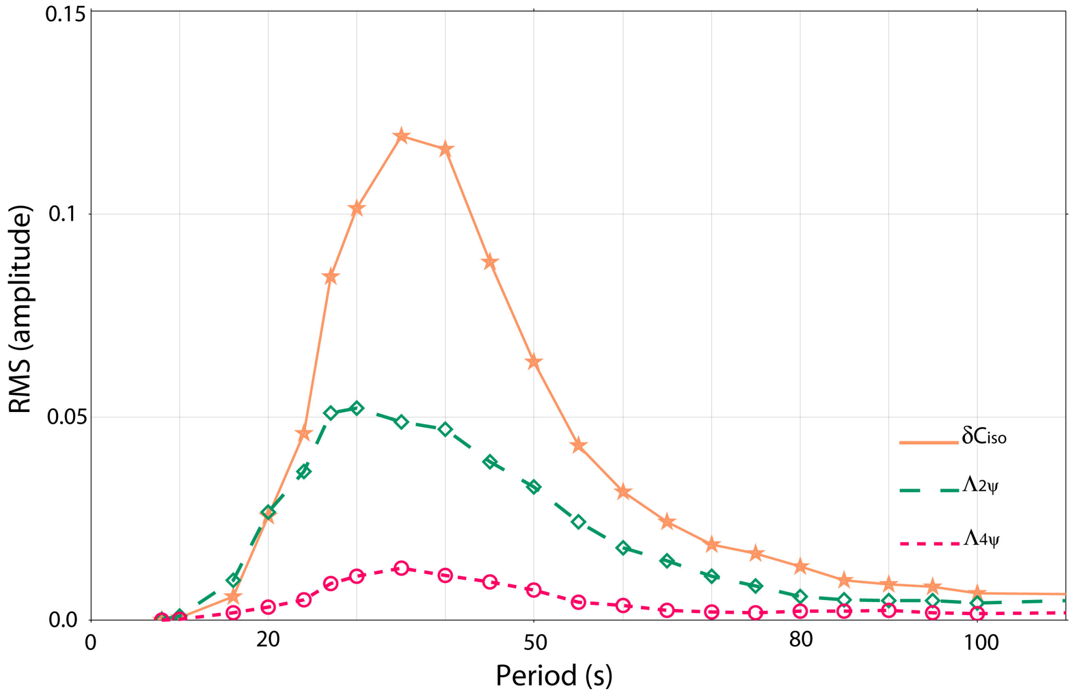

Figure S8. RMS of the amplitudes calculated for all periods for the isotropic terms (orange), the 2Ψ anisotropy (green) and the 4Ψ term (red). This figure is generated using the Generic Mapping Tools[29] and GIMP 2.6.

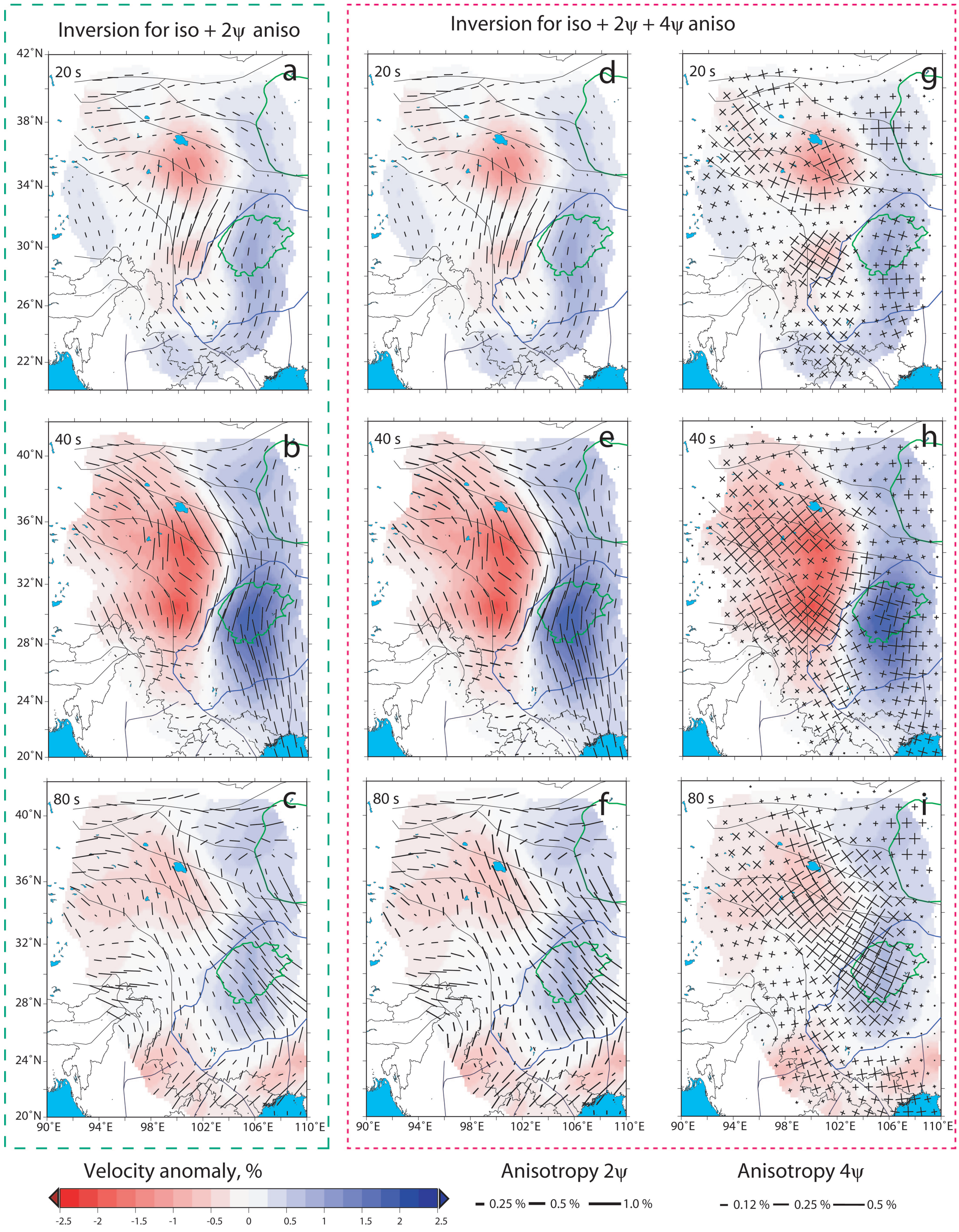

Figure S9. Models calculated while taking into account the 2Ψ anisotropy (a-c) and the 2Ψ and 4Ψ anisotropic terms (d-i). Panels(d-f) display the isotropic and 2Ψ anisotropy for this inversion, whereas panels (g-i) display the isotropic and 4Ψ anisotropy for this inversion. This figure is generated using the Generic Mapping Tools[29] and GIMP 2.6.

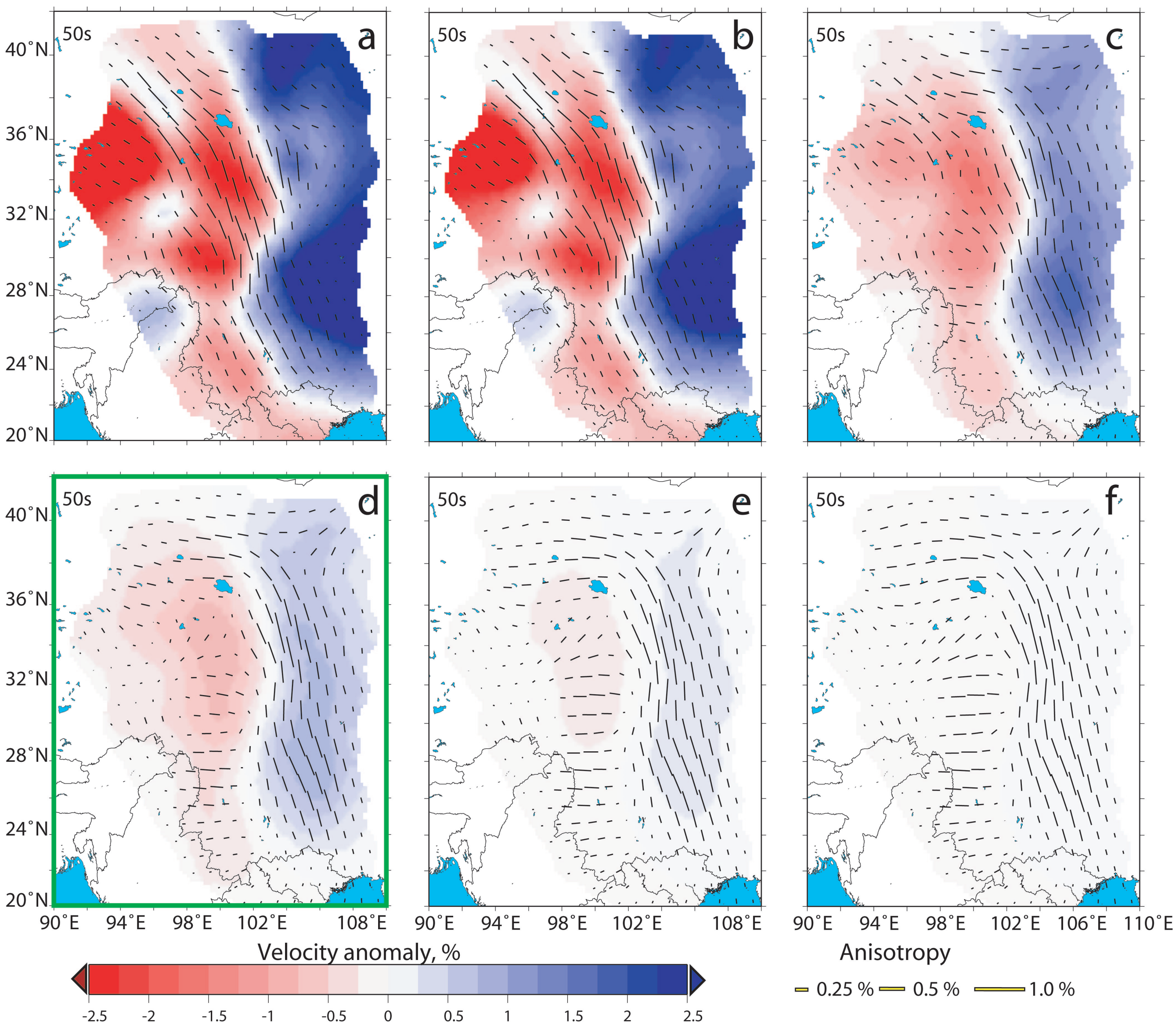

Figure S10. Effects of the isotropic damping parameter. The models have been obtained for a specific period of 50 seconds with increasing isotropic damping values from (a) to (f). All other parameters remain constant during the inversion process. The preferred model chosen for the final inversion is framed in green. This figure is generated using the Generic Mapping Tools[29] and GIMP 2.6.

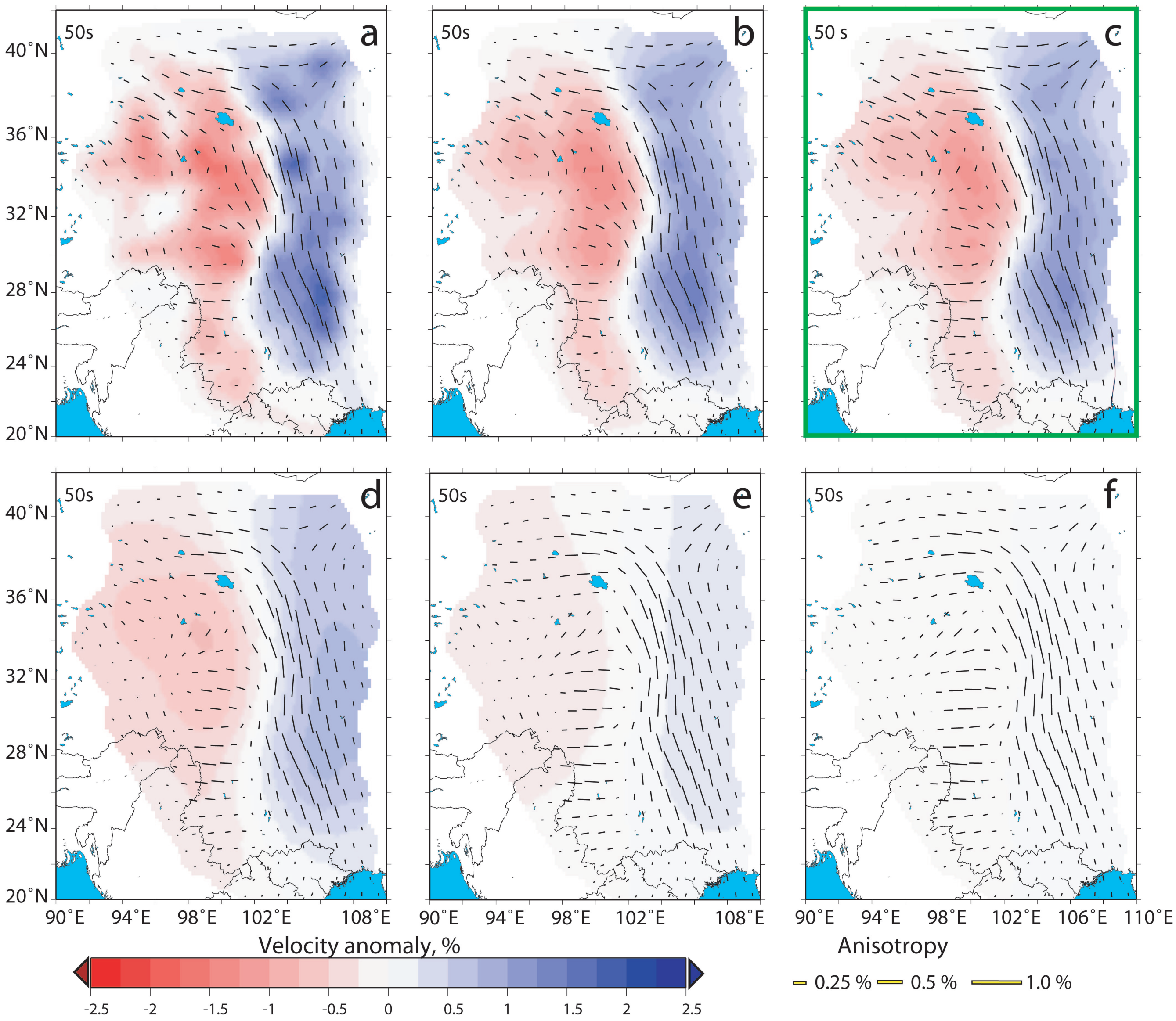

Figure S11. Effects of the isotropic smoothing parameter. The models have been obtained for a specific period of 50 seconds with increasing isotropic smoothing values from (a) to (f). All other parameters remain constant during the inversion process. The preferred model chosen for the final inversion is framed in green. This figure is generated using the Generic Mapping Tools[29] and GIMP 2.6.

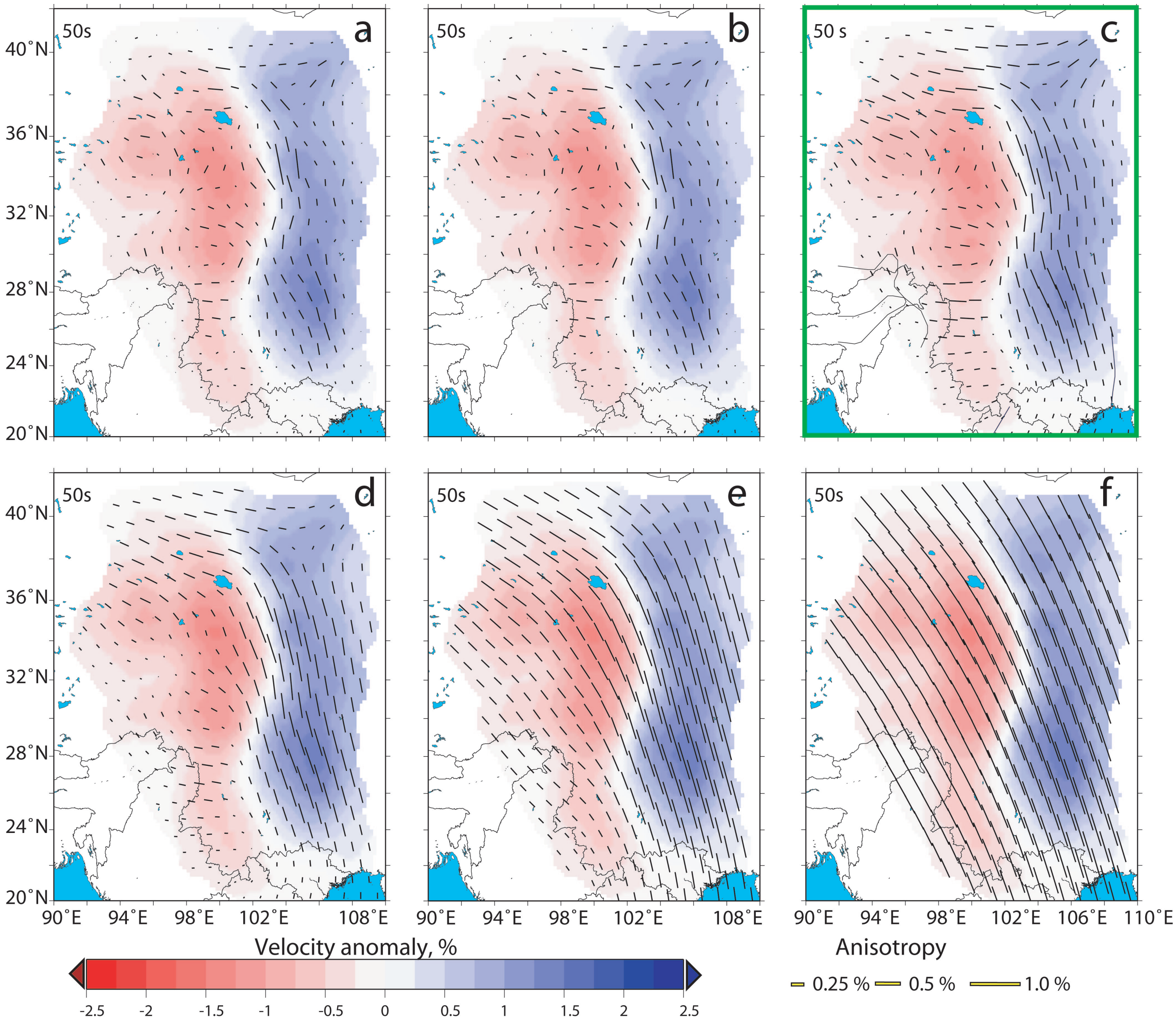

Figure S12. Effects of the anisotropic smoothing parameter. The models have been obtained for a specific period of 50 seconds with increasing anisotropic smoothing values from (a) to (f). All other parameters remain constant during the inversion process. The preferred model chosen for the final inversion is framed in green. This figure is generated using the Generic Mapping Tools[29] and GIMP 2.6.

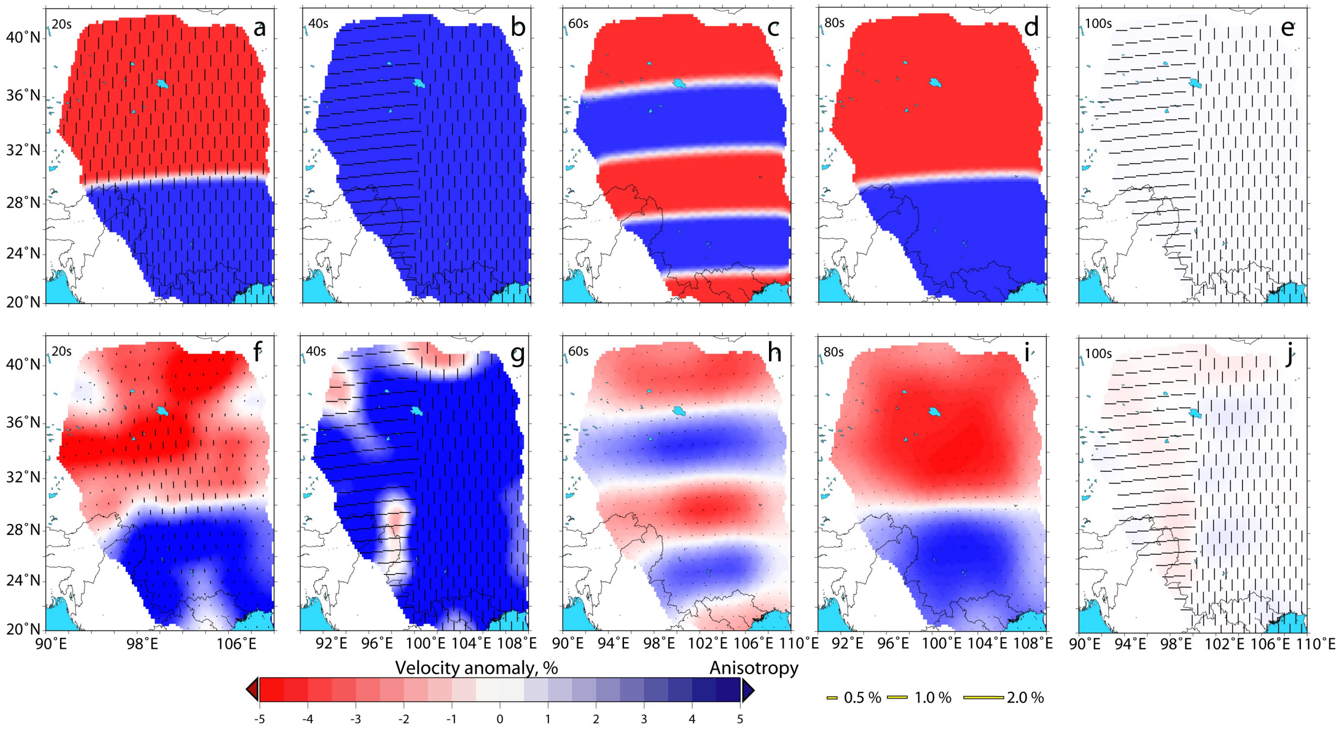

Figure S13. Resolution tests. (a-e) Input test models at periods of 20, 40, 60, 80 and 100 seconds; and (f-j) reconstructed models for respective periods. The reconstructions are performed to test the retrieval of both isotropic and anisotropic structures. This figure is generated using the Generic Mapping Tools[29] and GIMP 2.6.

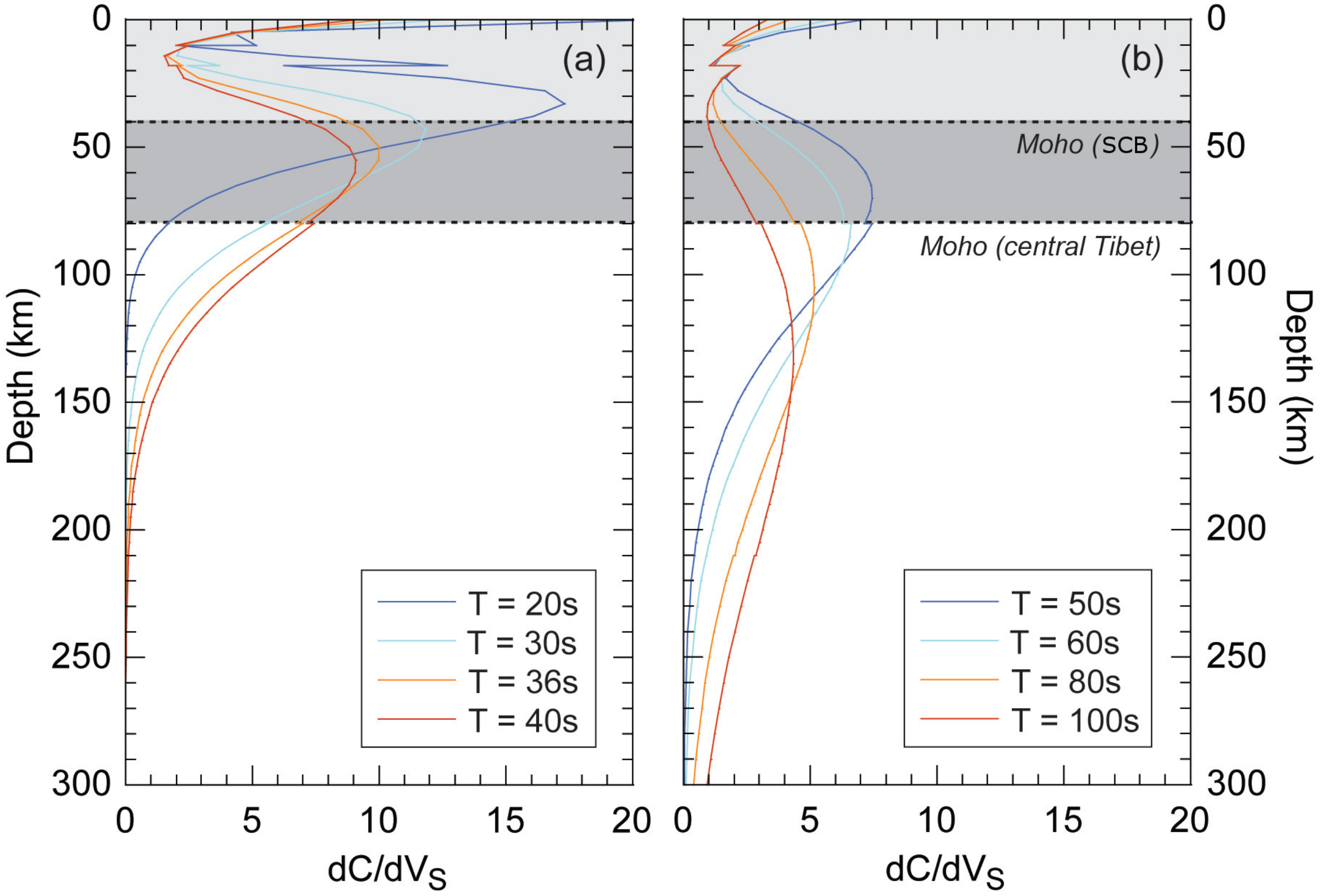

Figure S14. Sensitivities of the Rayleigh-wave phase velocities as functions of depth for selected periods. The model used to calculate the sensitivities is the continental AK135 model.[28] This figure is generated using the Generic Mapping Tools[29] and GIMP 2.6.

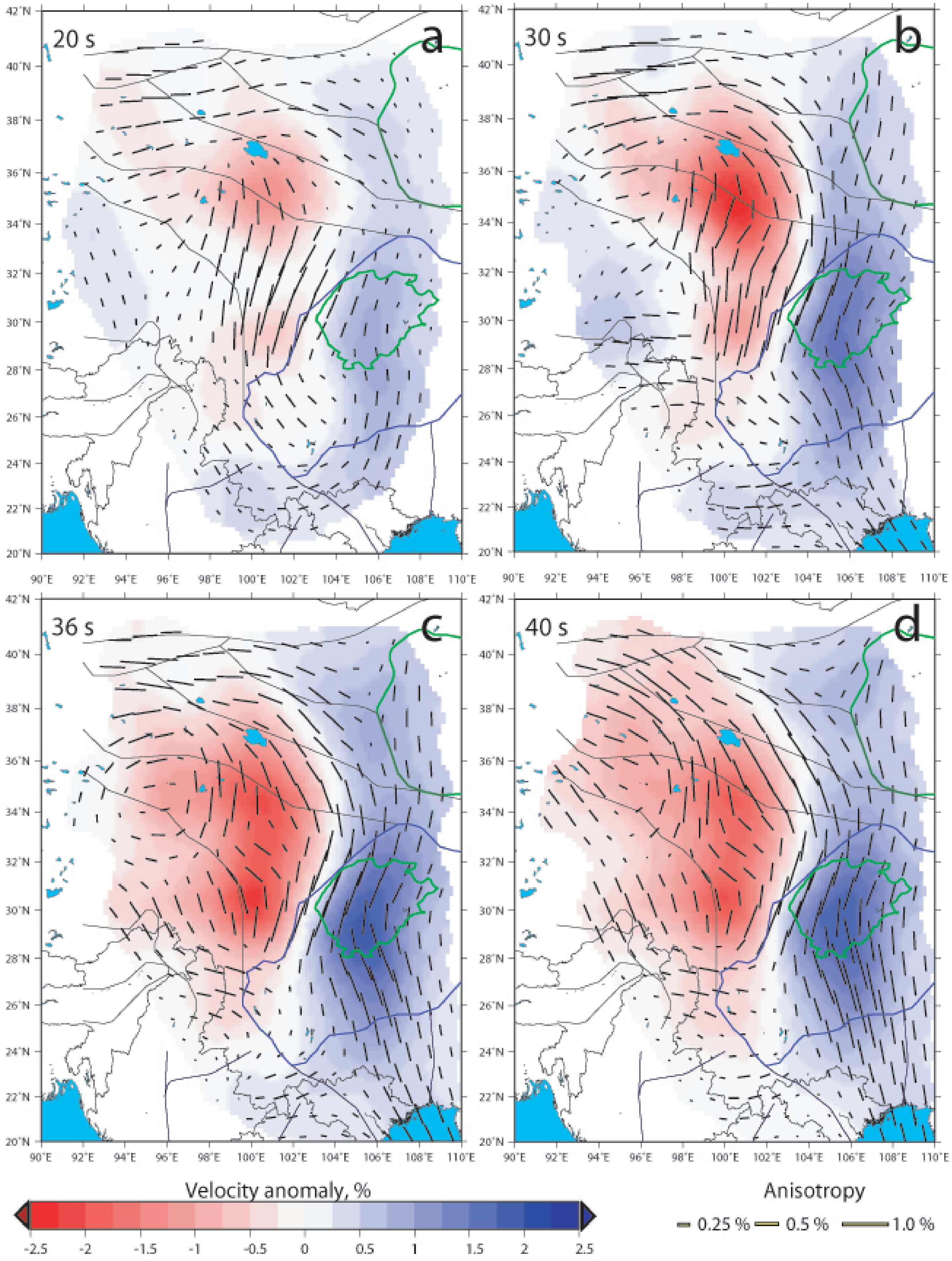

Figure S15. Maps of isotropic and azimuthal anisotropic anomalies in Rayleigh-wave phase velocity for specific periods. From (a) to (d) 20, 30, 36 and 40 seconds, respectively. This figure is generated using the Generic Mapping Tools[29] and GIMP 2.6.

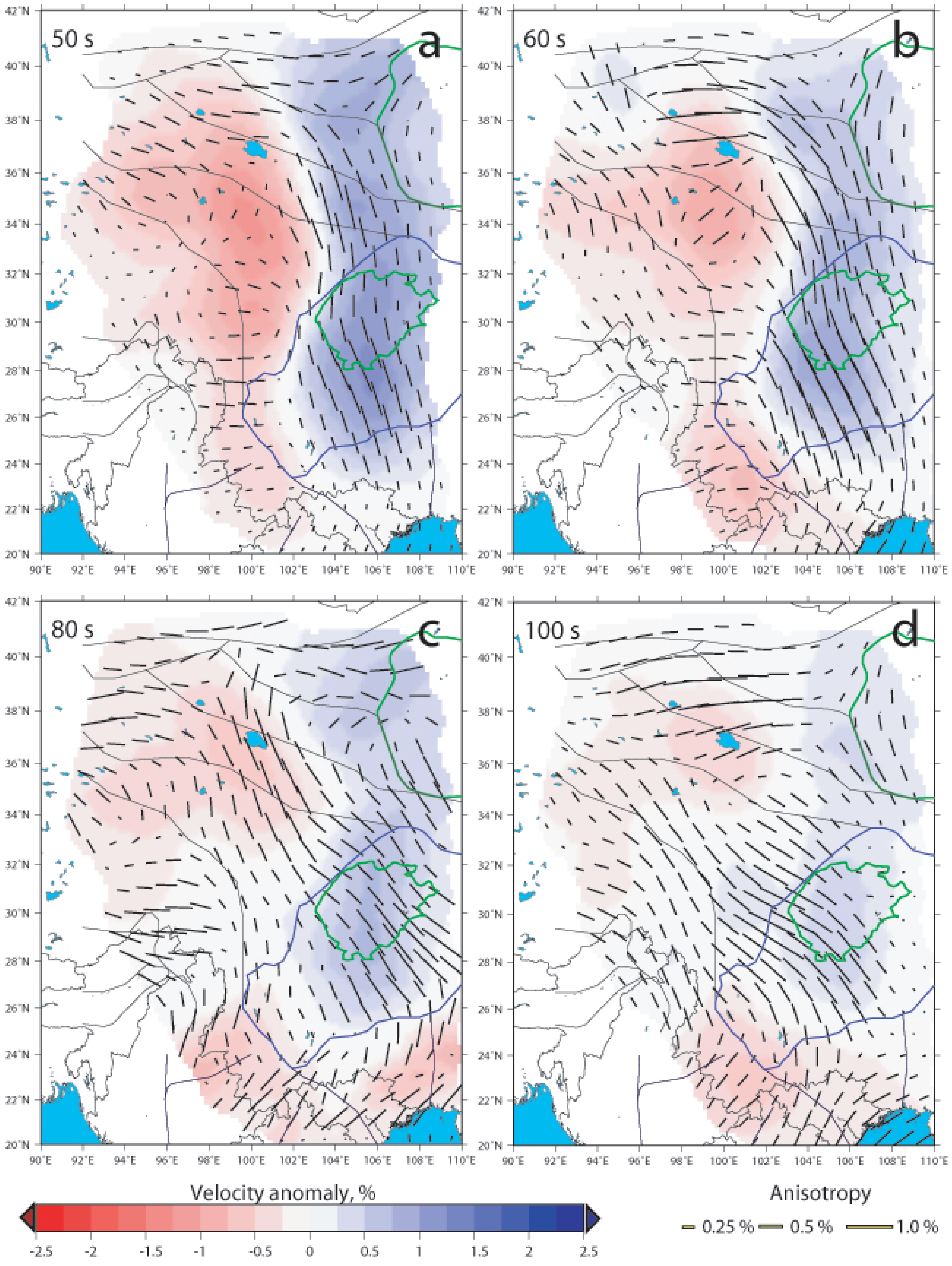

Figure S16. Maps of isotropic and azimuthal anisotropic anomalies in Rayleigh-wave phase velocity for specific periods. From (a) to (d) 50, 60, 80 and 100 seconds, respectively. This figure is generated using the Generic Mapping Tools[29] and GIMP 2.6.
